# Use of lipid-lowering agents is not associated with improved outcomes for tuberculosis patients on standard-course therapy: A population-based cohort study

**DOI:** 10.1371/journal.pone.0210479

**Published:** 2019-01-11

**Authors:** Yung-Tai Chen, Shu-Chen Kuo, Pei-Wen Chao, Yea-Yuan Chang

**Affiliations:** 1 Department of Medicine, Taipei City Hospital Heping Fuyou Branch, Taipei, Taiwan; 2 Institute of Clinical Medicine, School of Medicine, National Yang-Ming University, Taipei, Taiwan; 3 Division of Infectious Diseases, Department of Internal Medicine, Taipei Veterans General Hospital, Taipei, Taiwan; 4 National Institute of Infectious Diseases and Vaccinology, National Health Research Institutes, Miaoli County, Taiwan; 5 School of Medicine, Taipei Medical University, Taipei, Taiwan; 6 Department of Anesthesiology, Wan Fang Hospital, Taipei Medical University, Taipei, Taiwan; 7 Faculty of Medicine, School of Medicine, National Yang-Ming University, Taipei, Taiwan; 8 Division of Infectious Diseases, Department of Internal Medicine, National Yang-Ming University Hospital, Yilan, Taiwan; Management Sciences for Health, ETHIOPIA

## Abstract

**Objectives:**

Animal and ex vitro studies suggested lipid-lowering agents (LLAs) may be used as an adjunct to standard anti- tuberculosis (TB) treatment. No human study has been conducted to date. Using the Taiwan National Health Insurance Research Database (NHIRD), the current population-based cohort study sought to examine the association between use of LLAs and outcomes of patients with pulmonary TB receiving anti-TB treatment.

**Methods:**

Using a NHIRD from 2003 to 2010, this population-based cohort study retrospectively examined the association between LLAs (statins or fibrates) and the outcomes of patients with pulmonary TB receiving anti-TB treatment.

**Results:**

A total of 1452 adult patients newly diagnosed with pulmonary TB during the study period were identified and compared with 5808 matched patients. In the LAA cohort, 1258 received statin, and 295 received fibrate. Compared with patients who did not take LLA, patients who took oral LLAs had similar incidence of treatment completion at 9, 12, and 24 months.

**Conclusions:**

Neither statins nor fibrates provide clinical benefit superior to that achieved with standard anti-tuberculosis treatment. Future clinical trials should investigate the effects of statins and fibrates on short-course standard anti-TB therapy.

## Introduction

In 2016, tuberculosis (TB) affected 10.4 million people and claimed 1.7 million deaths worldwide [1]. Treatment outcomes for TB are unsatisfactory, as therapy is prolonged, complex, and often results in poor compliance [2]. Researchers have been searching for adjunctive therapies to use in combination with standard anti-TB therapy. Promising agents that are currently available include lipid-lowering agents (LLAs) [3]. Recent in vitro and animal studies showed that statins, alone or in combination with first-line anti-TB therapy, may reduce bacterial burden and enhance bactericidal activity [4]. Although the mechanism remains to be established, reduced levels of cholesterol in the cell membrane may reverse the arrest of phagosomal maturation induced by *Mycobacterium tuberculosis* [5]. Another type of LLA, fibrate, has been shown to inhibit intracellular growth of TB by inhibiting enoyl reductase [6] and inflammation caused by *Mycobacterium smegmatis* through a PPARα-independent pathway [7]. Clinical observation revealed the statin therapy was associated with a decreased risk of active TB [8]; however, no previous human study has been conducted in tuberculosis-infected patients. Using the Taiwan National Health Insurance Research Database (NHIRD) from Jan. 1, 2003 to Dec. 31, 2010, the current population-based cohort study sought to examine the association between use of LLAs and outcomes of patients with pulmonary TB receiving anti-TB treatment.

## Material and methods

The NHIRD contains demographic data, diagnoses according to the International Classification of Disease ninth revision (ICD-9-CM), procedures, and medications [9]. The Taiwan’s NHI program covers 99% of the population in 2004, and the coverage provides outpatient service and inpatient care. The Taiwanese Centers for Disease Control (TCDC) stipulate mandatory registry of newly diagnosed TB cases and monitoring of treatment. To encourage compliance with TCDC guidelines, physicians were rewarded with extra reimbursement through NHIRD if patients were diagnosed and treated according to TCDC guidelines. The reimbursement of claims related to a diagnosis of TB requires the positivity of microbiological tests or presence of pathological evidence typical of TB. This retrospective study included adult patients with new diagnoses of pulmonary TB (011.x) and double confirmation from NHIRD during the period from 2003–2010, the prescription of at least 2 anti-TB drugs (e.g., isoniazid, ethambutol, rifampin, pyrazinamide) for 2 months. Patients with age <20 years, a concomitant diagnosis of extra-pulmonary TB (010.x, 012.x–018.x), or antecedent TB were excluded. Patients were divided in two cohorts: in the LLA cohort, patients received fibrates or statins during the course of treatment for TB; members of the propensity-matched comparison cohort did not receive LLA. Each patient in the former group was matched with 4 patients in the comparison cohort with propensity score (±0.1SD) in terms of age (±5 years), sex, the year and month of index visit, monthly income, urbanization level, and comorbidities [10]. In order to ensure appropriate assessment of comorbid conditions as defined by ICD-9-CM codes, all patients included in the study were available for at least 3 years of follow-up [9]. The comorbidities included hypertension (ICD-9-CM: 401 and 405), diabetes mellitus (ICD-9-CM: 250), cerebrovascular disease (ICD-9-CM: 430–438), coronary artery disease (ICD-9-CM: 414), heart failure (ICD-9-CM: 428), myocardial infarction (ICD-9-CM:410), arrhythmia (ICD-9-CM: 427), dyslipidemia (ICD-9-CM: 272), chronic kidney disease (ICD-9-CM: 585), and chronic liver disease (ICD-9-CM:571). The primary outcome was the incidence of treatment completion at 9, 12, and 24 months after anti-TB drugs were administered. Some patients used stain and fibrate sometimes. Treatment completion was confirmed by the reimbursement to physicians from the NHIRD for compliance with TCDC policy. All subjects were followed until death, loss to follow-up, completion of treatment, or two years after initiation of anti-TB drugs.

Baseline demographic characteristics between the patients with and without LLA user were compared by use of the Pearson χ^2^ test for categorical variables and the independent *t* test for parametric continuous variables. The incidence rates of tuberculosis treatment completion at 9, 12, and 24 months and control cohort were calculated from Poisson distribution. We used Cox regression models to calculate crude and adjusted hazard ratios (HRs) and 95% confidence intervals (CIs) of tuberculosis treatment completion at 9, 12, and 24 months and control cohort.[11] The SQL Server 2012 (Microsoft Corp, Redmond, WA) was used for data linkage, processing, and sampling. Calculation of Propensity scores was performed with SAS version 9.3 (SAS Institute, Cary, NC). Other statistical analyses were conducted with STATA statistical software (version 12.0; StataCorp, College Station, TX). A P value <0.05 was considered statistically significant.

The study was reviewed and approved by the institutional review board of National Health Research Institute [EC1041007-E]).

## Results

**Fig 1:**
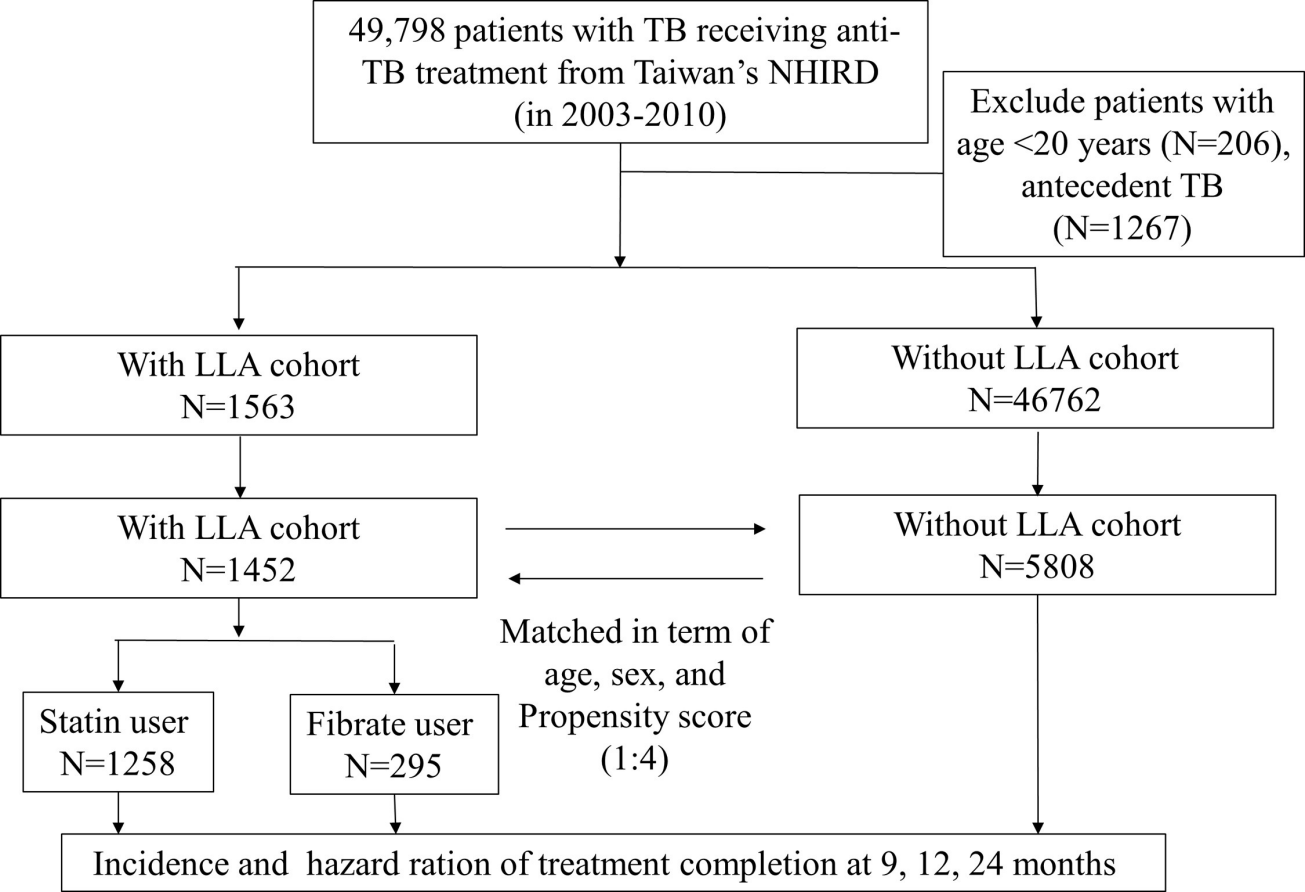
Overall framework of the research design and sampling strategy. NHIRD: National Health Insurance Research Database; TB:tuberculosis; LLA: lipid lowing agent.

A total of 1452 adult patients newly diagnosed with pulmonary TB were identified; 5808 matched patients were included in the comparison cohort (Fig 1). Mean age in the LLA cohort was 65 years. The majority of LLA patients were male (69.9%), and had ≥ 4 comorbidities (64.7%). Dyslipidemia (83.5%) was the most common comorbid disease, followed by hypertension (78.5%) and diabetes mellitus (72.3%). There was higher prevalence rates of myocardial infarction (8.6% vs. 7.0%) in the patients with LLA cohort compared to those without LLA. (Table 1)

**Table 1:** Characteristics of patients with newly diagnosed pulmonary tuberculosis.

Characteristics	Lipid lowing agent user	Matched control	*P* value
**Patient No.**	1452	5808	
**Age, mean (SD), years**	65.1 (12.5)	65.3 (12.4)	0.581
**Male sex**	1,016 (69.9%)	4,064 (69.9%)	1.000
**Monthly income**			0.933
** Dependent**	448 (30.8%)	1,837 (31.6%)	
** 0–19 100 NT dollars**	334 (23%)	1,306 (22.4%)	
** 19 100–42 000 NT dollars**	600 (41.3%)	2,395 (41.2%)	
** > 42 000 NT dollars**	70 (4.8%)	270 (4.6%)	
**Urbanization**^a^			0.962
** Level 1**	747 (51.4%)	3,009 (51.8%)	
** Level 2**	578 (39.8%)	2,273 (39.1%)	
** Level 3**	110 (7.5%)	455 (7.8%)	
** Level 4**	17 (1.1%)	71 (1.2%)	
**Charlson comorbidity index score**^b^			0.072
** 0**	26 (1.7%)	55 (0.9%)	
** 1**	109 (7.5%)	404 (6.9%)	
** 2**	177 (12.1%)	698 (12%)	
** 3**	200 (13.7%)	849 (14.6%)	
** ≥ 4**	940 (64.7%)	3,802 (65.4%)	
**Using lipid lowering agents**			
** Statins**	1208	-	
** Fibrates**	295	-	
**Comorbid disease**			
** Hypertension**	1,141 (78.5%)	4,582 (78.8%)	0.796
** Diabetes mellitus**	1,050 (72.3%)	4,299 (74%)	0.187
** Cerebrovascular disease**	527 (36.2%)	2,062 (35.5%)	0.573
** Coronary artery disease**	765 (52.6%)	2,998 (51.6%)	0.467
** Heart failure**	297 (20.4%)	1,092 (18.8%)	0.152
** Myocardial infarction**	126 (8.6%)	411 (7.0%)	0.037
** Arrhythmia**	525 (36.1%)	2,108 (36.2%)	0.922
** Dyslipidemia**	1,213 (83.5%)	4,875 (83.9%)	0.714
** Chronic kidney disease**	398 (27.4%)	1,602 (27.5%)	0.895
** Chronic liver disease**	557 (38.3%)	2,337 (40.2%)	0.191
**Propensity score (SD)**	0.10 (0.06)	0.10 (0.06)	0.978

SD: standard deviation; NT$: new Taiwan dollars.

^a^Urbanization levels in Taiwan are divided into four strata according to the Taiwan National Health Research Institute publications. Level 1 designates the most urbanized areas, and level 4 designates the least urbanized areas.

^b^Charlson Comorbidity Index (CCI) score is used to determine overall systemic health. With each increased level of CCI score, there are stepwise increases in the cumulative mortality.

In the LLA cohort, 1258 patients received statins, and 295 received fibrates. Compared with patients who did not take LLA, patients who took LLAs showed no increase in the rate of treatment completion (Table 2). The incidence of treatment completion was similar at 9, 12, and 24 months (incidence 1.16 vs. 1.11, adjusted hazard ratio [aHR] 1.04, 95% confidence interval [CI] 0.96–1.12 at 9 months; incidence 1.06 vs. 1.02, aHR 1.04, 95% CI 0.96–1.11 at 12 months; and incidence 0.71 vs. 0.69, aHR 1.03, 95% CI 0.97–1.11 at 24 months).

**Table 2:** Incidence and hazard ratio of among tuberculosis patients.

					Propensity Score–Matched			
					Crude		Adjusted ^a^	
	Number of patients	No. of Event	Person-years	Incidence rate*	HR (95% CI)	*P*	HR (95% CI)	*P*
*9 months*								
**Control cohort**	2116	3361	2931	1.15	As Reference		As Reference	
**Using LLA**	555	865	746	1.16	1.04 (0.96–1.12)	0.319	1.04 (0.96–1.12)	0.309
** Statin**	459	721	621	1.16	1.04 (0.95–1.12)	0.398	1.04 (0.96–1.12)	0.384
** Fibrate**	111	164	143	1.15	1.04 (0.88–1.24)	0.616	1.04 (0.88–1.24)	0.648
*12 months*								
**Control cohort**	1825	3508	3430	1.02	As Reference		As Reference	
**Using LLA**	479	930	877	1.06	1.04 (0.96–1.11)	0.335	1.04 (0.96–1.11)	0.324
** Statin**	398	773	730	1.06	1.03 (0.95–1.12)	0.424	1.03 (0.96–1.12)	0.409
** Fibrate**	94	178	169	1.06	1.04 (0.89–1.23)	0.603	1.04 (0.88–1.23)	0.640
*24 months*								
**Control cohort**	1622	3565	5140	0.69	As Reference		As Reference	
**Using LLA**	440	943	1332	0.71	1.03 (0.96–1.11)	0.370	1.03 (0.97–1.11)	0.359
** Statin**	365	784	1108	0.71	1.03 (0.95–1.12)	0.463	1.03 (0.95–1.12)	0.446
** Fibrate**	87	181	258	0.70	1.05 (0.89–1.23)	0.572	1.04 (0.89–1.23)	0.610

HR: hazards ratio; CI: confidence interval; LLAs: lipid-lowering agents.

* Per person-years.

^a^ Adjusted for propensity score.

## Discussion

The protective effect of statins, as either monotherapy or adjunctive therapy, against TB has been demonstrated [12]. Despite the lack of a direct anti-mycobacterial effect, statins reduce bacterial burden by reducing cholesterol accumulation within *Mycobacterium tuberculosis* and enhancing autophagy and phagosomal maturation [5]. These effects may be mediated by the anti-inflammatory response and immunomodulatory effects [12]. Clinical studies have confirmed that statin treatment is associated with a lower incidence of active TB [3, 8]. Recent studies have demonstrated their use as an adjunct in combination with standard therapy. Animal experiments showed statins enhance the bactericidal effect of standard therapy [4] and shorten the duration required to sterilize TB [13]. However, the results of our study failed to confirm the role of statins as adjunctive therapy to standard anti-TB treatment. Prolonged first-line treatment with anti-TB drugs is often successful in treating human patients, which may mask the adjunctive effect of statins. A mouse study also showed that after 4.5 months of anti-TB therapy, simvastatin provided no additional benefit when combined with standard therapy [13]. However, when treatment duration was decreased to 2.5 months, combination of simvastatin with standard therapy decreased the relapse rate of TB, compared to standard therapy alone. A prospective study comparing a standard course of anti-TB drugs with a short course of TB drugs in combination with LLAs may be warranted.

The strength of our study included propensity score-matching for multiple comorbid diseases. Nonetheless, our retrospective study has some limitations. First, this study was limited by the small number of patients who took fibrates during pulmonary TB infection. Second, treatment course may have been prolonged because of bacterial resistance or side effects of drug treatment. However, multidrug resistant strains of TB represented only 1.2% of new-onset TB cases in Taiwan [14]. Third, the beneficial effect of LLAs may require chronic use (>90 days) [8]. The duration of statin use may have affected protection against TB [8, 15]. We did not systematically study accumulated dose in this study.

In the era of end TB strategy, we need new therapy or adjunctive therapy to shorten TB treatment. It is important to find therapeutic agents with which rate of treatment completion for TB infection can be enhanced in public health. We have yet to find the beneficial effects of statin with current anti-TB therapy, a prospective clinical trial is needed to show it in future.

## Conclusion

In conclusion, the results presented above do not support an adjunctive effect of LLAs in combination with standard anti-TB treatment. Further clinical trials are needed to investigate the adjunctive effect of statin and fibrate in the treatment of TB.
